# Ambulatory health service users' experience of waiting time and expenditure and factors associated with the perception of low quality of care in Mexico

**DOI:** 10.1186/1472-6963-10-178

**Published:** 2010-06-23

**Authors:** Alma Lucila Sauceda-Valenzuela, Veronika J Wirtz, Yared Santa-Ana-Téllez, Maria de la Luz Kageyama-Escobar

**Affiliations:** 1 Center for Health Systems Research, National Institute of Public Health, Mexico; 2 Center for Evaluation and Survey Research, National Institute of Public Health, Mexico

## Abstract

**Background:**

A principal reason for low use of public health care services is the perception of inferior quality of care. Studying health service user (HSU) experiences with their care and their perception of health service quality is critical to understanding health service utilization. The aim of this study was to define reference points for some aspects of health care quality and to analyze which HSU experiences resulted in perceptions of overall low quality of care.

**Methods:**

Data from the National Health Survey 2006 were used to compare the experiences of HSUs with their ambulatory care at Ministry of Health and affiliated institutions (MOH), social security institutions (SSI) and private institutions (PrivI). Reference points of quality of care related to waiting time and expenditure were defined for each of the three types of institutions by analyzing HSU experiences rated as 'acceptable'. A multivariable logistic regression model was used to identify the principal factors associated with the general perception of low quality of care.

**Results:**

A total of 11,959 HSUs were included in the analysis, of whom 37.6% (n = 4,500) HSUs received care at MOH facilities; 31.2% (n = 3,730) used SSI and 31.2% (n = 3,729) PrivI. An estimated travel and waiting time of 10 minutes respectively was rated as acceptable by HSUs from all institutions. The differences between the waiting time rated as acceptable and the actual waiting time were the largest for SSI (30 min) in comparison to MoH (20 min) and PrivI (5 min) users. The principal factors associated with an overall perception of low quality of care are type of institution (OR 4.36; 95% CI 2.95-6.44), waiting time (OR 3.20; 95% CI 2.35-4.35), improvement of health after consultation (OR 2.93; CI 2.29-3.76) and consultation length of less than 20 minutes (2.03; 95% CI 1.60-2.57).

**Conclusions:**

The reference points derived by the HSUs' own ratings are useful in identifying where quality improvements are required. Prioritizing the reduction of waiting times and improving health status improvement after consultation would increase overall quality of care ratings.

## Background

Health service users (HSUs) have a legitimate interest in the provision of health care with a high level of quality as they are financial contributors, tax paying citizens and recipients of care [1]. However, public health services, particularly in developing countries, struggle to provide not only a high technical quality of care but also responsiveness to non-medical expectations [2,3]. As a result many HSUs, particularly in developing countries, prefer using fee-for-service care with high out-of-pocket expenditures. Hence, studying perceptions of quality of care is critical to understanding health service utilization.

Because HSU perception of health services quality is a result of two principal factors: first, their experiences with access to care and use and second, the respective ratings or value they assign to these experiences, it has been argued that it is important to analyze both experiences and ratings simultaneously [4-7]. Reported experiences with access to health services include, among other aspects, transport time to the health service facility and the waiting time. Experiences with use include for example the physical examination by the physician, information provided by the health care professional to the user and the length of consultation. The ratings include, for instance, how long the HSU perceived the travel time to be, waiting time at the facility (e.g. long or very long) and how they rate the information they received (very adequate or adequate). Many previous studies in Latin America have focused only on patient's satisfaction (e.g. the % of patients satisfied with the waiting time) without taking into consideration the actual experience (e.g. length of waiting time) of access to and use of services [8-11]. The evaluation of patient's satisfaction does not necessarily mean measuring of patient's experiences as "satisfaction involves a cognitive evaluation of and emotional reaction to health care" [12] Hence, Coulter [7] has argued that that it is central to measure patients' experience in combination with their respective ratings. Information on both of these aspects is essential to improving quality in health services, as they can provide important reference points against which HSU experiences can be measured.

As in other Latin America countries, insufficient quality of services and user dissatisfaction with the public and social security services have been cited as two of the main reasons why people in Mexico opt to use fee-for-services despite its financial implications. The use of fee-for-services among insured and uninsured population has increased over the last years in Mexico. In 2000 31.1% used fee-for-service [13], meanwhile in 2005 37.6% reported using them [14]. Fee-for-service care has been receiving the highest overall user satisfaction in recent years [15,13]. To tackle insufficient quality of care, over the last decades the Mexican government has launched various initiatives to improve quality of care. Since 2007, the Comprehensive Quality System (Sistema Integral de Calidad or SICalidad) has aimed at quality improvement in the public sector. To make providers more accountable, HSU satisfaction with quality of services is reported periodically. For instance, in 2006 an average of 98% of ambulatory care patients were satisfied with the information received from the physician and 89% with the prescription filling [16]. However, the data reported do not identify what experiences resulted in 11% of the HSUs reporting that they are not satisfied with their prescription filling (e.g. partial prescription filling, receiving no medicines or receiving inadequate information).

Previous studies on user satisfaction with the quality of care comparing different service providers in Mexico reported that the primary reasons for perceived low quality were long waiting times and poor clinical examination [15]. Other studies have focused on comparing interpersonal quality of care between different health care providers in Mexico, which found that ambulatory health service HSUs were most frequently unsatisfied with the waiting times whereas hospitalized health service HSUs most frequently mentioned limited choice of provider as the reason for dissatisfaction [17]. A recent study by Puig et al [18] focused on which health service user characteristics are associated with the perception of overall good quality of health care and found that age, health status and education were associated with the overall perception of health care quality. Although these studies provide insight into the reasons *why *HSUs perceived the overall quality as low [15,17] or *what HSU characteristics *influence the perception of good quality of care [18] they do not provide information on which HSU experiences specifically resulted in a low quality rating and therefore, need to be improved. In other words, to give some examples, we need to know the average waiting time a HSU would rate as acceptable and what choice of providers is most frequently rated acceptable. To improve health quality programs in Mexico and other countries this information is of high relevance. In addition, there is a paucity of information about which aspects of the experience of care most influenced the general perception of low quality when adjusting for patient characteristics. Such information would allow prioritizing in programs most relevant to HSUs. Therefore, the objectives of the present study were twofold: first to identify, on the bases of HSU experiences and their respective ratings of waiting time and expenditure on health care, reference points of acceptable quality and second, to analyze which HSU experiences are associated with a general perception of low health care quality.

## Methods

The present study is based on an analysis of the most recent (2006) National Health and Nutrition Survey (ENSANUT) which has been carried out every six years since 1988 and explores the characteristics of household members in terms of health, physical activity, diet, chronic diseases and health service utilization [19]. The 2006 survey is a nationally representative, multistage, stratified sample of 47,152 households for which information was collected between October 2005 and May 2006 [19]. (A rejection rate of approximately 15% has been taken into account when calculating the sample size.) For analysis purposes, information from the household and health ambulatory service utilization portion was used. In Mexico ambulatory health services (also called outpatient services) are defined as all diagnostic, curative and preventative services provided to individuals in and outside the hospital who depart after service delivery. This excludes hospital services (also called inpatient services) which are defined as those where individuals receive diagnostics or curative services and stay for longer than 24 hours at the service delivery site (clinic or hospital). The HSU experiences instrument of ENSANUT has been used in earlier national health services 1994 and 2000. A study testing for reliability and validity was carried out prior to their use [15]. The different aspects that the instruments include to analyze HSU experience are based on the literature of measuring quality of care [20,21]. A random sample of household members was asked in a face-to-face interview about health service utilization in the previous 14 days and their experiences and perception in access and use of health services.

HSUs were stratified according to the three main health care providers in Mexico: (1) Those attending Ministry of Health and related institutions (MOH) which comprises the Ministry of Health, Seguro Popular, Comprehensive Family Development Program, Red Cross, Civil Hospital, National Institutions, and the social program Oportunidades; (2) Social Security Institutions (SSI) including the Mexican Institute of Social Security (IMSS), the Institute of Social Security and Services of State Workers (ISSSTE), Military Services (MARINA/DEFENSA), and Mexican Petrol (PEMEX); and (3) Private Institutions (PrivI). The MOH and affiliated institutions provide care to the uninsured population; the SSI provides care to formal sector employees and their families and the private sector offers services on a fee-for-service base [22]. To uninsured individuals, the MOH provides services free of charge only for hospitalized patients; medicines prescribed in ambulatory care (outpatient) are subsidized for uninsured individuals. Although according to regulations, fees are inversely related to patients' income, [22] in practice, it varies between and within states whether fees are applied or not. Recently the Ministry of Health has started to provide care for those affiliated with Seguro Popular, a new government program which offers basic health services to those who were previously uninsured [23]. Seguro Popular purchases services from different providers, in most Mexican states via the MOH. Those with Seguro Popular have the right to receive a defined list of services and essential medicines free at the point of care. In this context it is relevant to note that, although services at the SSI in Mexico are pre-paid and free of charge at the point of care, there are circumstances (e.g. lack of supply) in which HSUs of SSI do not obtain their medicines or laboratory tests at the institution. In these cases the HSUs have to pay for them using outside private pharmacies or clinical laboratories. In exceptional cases, patients who are not affiliated with SSI are offered care but they are charged for the received services.

Using HSU ratings of experiences in accessing and using health services, reference points of acceptable quality of care related to some aspects of care -namely travel and waiting time as well as expenditure- were developed. This process was conducted as follows: HSUs were asked to rate travel and waiting time ('very short', 'short', 'regular', 'long' and 'very long'), as well as expenditure on medicines, laboratory tests and consultation ('very cheap', 'cheap', 'regular' 'expensive' and 'too expensive'). The answers 'very short' and 'short' as well as 'cheap' and 'very cheap' were considered to indicate acceptable quality. Then the median travel and waiting time as well as median expenditure for medicines, laboratory tests and consultation reported as acceptable were calculated for each of the three institution types (MOH, SSI, and PrivI). These values were defined as reference points for acceptable quality of care and were compared against the reported time of travel, waiting and expenditure on medicines, laboratory tests and consultation for each type of institution.

Then we analyzed HSU experiences with aspects of quality of care. In order to permit more systematic reporting of these quality-related aspects we divided them into four areas: access, structure, process and outcome (adopted from Donabedian [20] who distinguished among structure, process and outcome) and from Anderson [21] whose work focuses on accessibility to health care. (i) Access includes HSU estimated travel time to the health facility; the estimated waiting time at the facility; expenditure on medicines, laboratory tests, and consultation (amount of expenditure reported in Mexican pesos); (ii) Structure comprises prescription filling, receiving laboratory tests and whether or not the HSU had to pay for the consultation (binary in yes or no); (iii) Process includes the estimated duration of consultation time and whether the HSU received a referral for laboratory tests and prescription for medicines; and (iv) Results including reported improvement in health status after service use.

Finally, we analyzed which HSU experiences resulted in an overall perception of low quality of care. We conducted a multivariable binominal logistic regression analysis with the rating of low quality of care being the dependent variable. Initially, overall perception of quality of care was measured on a scale from 1 to 5, where 1 was very high and 5 very low quality. For the purpose of this study the variable was dichotomized as low (3 to 5) with a value of 1 and high quality rating (1 and 2) with a value of zero. In a first step, we included in the model all variables reported in the literature as associated with low quality of care ratings [5,24]. In addition, those variables were also included which the authors on the basis of the questionnaire identified as relevant. The variables included were: socio-demographic characteristics of HSU (age, education, sex, socioeconomic status, degree of marginalization, region, type of location (urban rural), social security affiliation, employment -yes or no), reason for health service use (acute disease, chronic disease or prevention), length of illness, perceived severity of illness, health care provider (physician, nurse or other health care professional), improvement of health status after consultation, self-reported health status at the time of the survey (good, regular, bad), reported experience of access and health service use (travel and waiting time, length of consultation; charges for consultation, medicines, laboratory tests) and type of institution (MOH, SSI or PrivI).

Additionally, we conducted the same multivariable analysis for each of the institution types to identify possible interactions between the overall rating of quality of care and type of health care institution.

The National Health and Nutrition Survey received approval by the Ethics Committee of the National Institute of Public Health in 2005.

## Results

In total, 11,959 HSUs were included in the analysis of whom 37.6% (n = 4,500) HSUs received care at MOH, 31.2%; (n = 3,730) used SSI and 31.2% (n = 3,729) PrivI. About 32% of those HSUs with SSI affiliation decided to use PrivI instead of SSI where they would receive free services at the point of care.

**Figure 1:**
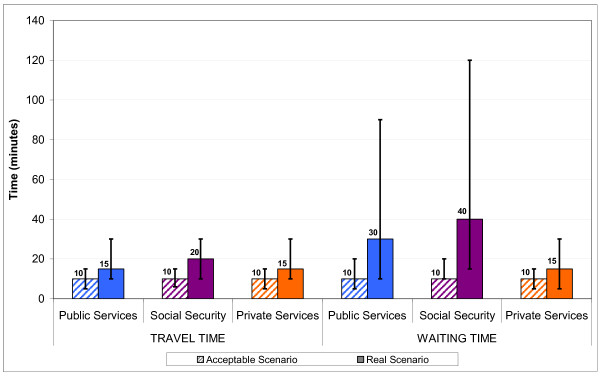
**Comparison of acceptable and reported travel and waiting time (in minutes)**. Note: Bars mark the interquartile range.

**Figure 2:**
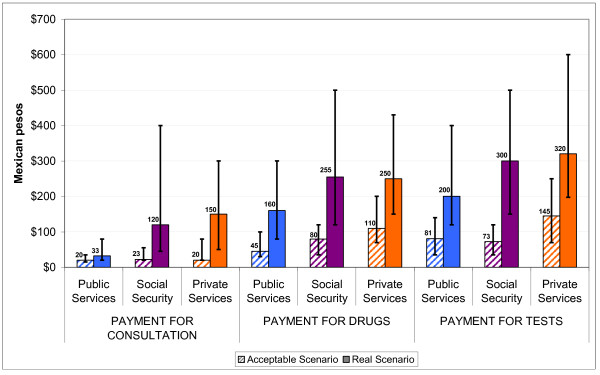
**Acceptable and reported payment of consultation, medicines and laboratory tests (in Mexican pesos)**. Note: Bars mark the interquartile range. Only health services users who had to pay for services were included in this analysis

Comparing the reference points for travel and waiting times that HSUs defined as acceptable shows that there were no differences between institutions (Figure 1). An estimated average travel and waiting time of 10 minutes respectively was rated as acceptable by HSUs from all three institution types. When comparing acceptable waiting time with those reported shows that the largest difference exists for HSUs of SSI with 30 minutes followed by HSUs of MOH with 20 minutes and those of PrivI with only a 5 minute difference. Figure 2 shows that acceptable amount of expenditures on consultation, medicines and laboratory tests vary between institutions (note that only health services users who had to pay for services were included in this analysis). The difference between reported and acceptable median expenditure on medicines differed by 3.6 times ($160 versus $45) for HSUs of MOH and 2.3 times ($250 versus $110) for HSUs of PrivI.

The experiences of HSUs to access and use of services are reported in Table 1. The median travel time differs 5 minutes between institution types. HSUs using PrivI had to pay more frequently for medicines, consultation and laboratory tests compared to HSUs of MOH; HSUs of SSI paid least frequently. With respect to process related aspects, HSUs of SSI reported the shortest estimated consultation time (15 minutes) compared to MOH and PrivI (20 minutes respectively). Fewer HSUs of SSI reported that their health status after consultation had improved in comparison to HSUs of MOH and PrivI (70.4% versus 75.4% and 87.1% respectively).

**Table 1:** Health service users' (HSU) experiences in access and use according to the institution of care and overall perception of quality of care

Variable	All institutions	HSU of public institutions (MOH)	HSU of social security institutions (SSI)	HSU of private institutions (PrivI)	p value*
	**n = 11,959**	**n = 4,500**	**n = 3,730**	**n = 3729**	

**Access**					

Travel time	20 (10-30)	15 (10-30)	20 (10-30)	15 (10-30)	<0.001

Waiting time	30 (10 - 60)	30 (10-90)	40 (15 - 120)	15 (5 - 30)	<0.001

Expenditure on medicines**^+^	$200 ($120 - $400)	$150 ($75 - $300)	$250 ($100 - $500)	$250 ($150 - $416)	<0.001

Expenditure on laboratory^+ ^tests	$300 ($150 - $500)	$205 ($120-$400)	$300 ($150-$500)	$320 ($198-$600)	<0.001

Expenditure on consultation^+^	$100 ($25 - $250)	$32.5 ($20 - $80)	$120 ($45-$400)	$150 ($50-$300)	<0.001

**Structure**					

% of HSU with prescription filled	66.3	64.2	90.3	45.6	<0.001

% HSU paying for their medicines	45.8	36.8	6.6	89.0	<0.001

% HSU which had their laboratory tests	75.6	71.8	77.1	77.6	<0.001

% HSU which paid for their laboratory tests	32.22	40.54	5.51	63.46	0.006

% HSU which paid for their consultation	36.7	27.0	2.4	87.1	<0.001

**Process**					

Duration of consultation	20 (15 - 30)	20 (10 - 30)	15 (10 - 25)	20 (15 - 30)	<0.001

% HSU who received a prescription	86.5	82.3	86.1	92.7	<0.001

% HSU who received a prescription for laboratory tests	24.7	19.7	34.2	22.4	<0.001

**Results**					

% HSU who reported improved health status after their consultation	77.6	75.4	70.4	87.1	<0.001

% HSU who perceived the overall quality of care low	18.34	18.1	28.3	8.7	<0.001

Values are medians or percentages, percentile 25-75 in parentheses; *p value of Kruskal Wallis differences between the three types of services; **Expenditure reported in Mexican pesos of 2006; +out of those HSU who paid

The overall perception of low quality of care was highest for HSUs of SSI (28.3%) followed by HSUs of MOH (18.1%) and lowest for HSUs of PrivI (8.7%) (Table 1). The independent variables that best fit the final models were: age, self-reported health status, reason for health service use, improvement of health status after the consultation, waiting time, consultation length, payment of consultation, prescription filling and institution of care (Table 2). The most relevant aspects determining the perception of a low quality of care was the institution type: the HSUs of MOH and SSI rated the quality of care lower than HSUs of PrivI (OR 4.36 for SS and 2.38 for MOH in comparison to HSUs of PrivI). A waiting time of longer than 60 minutes and consultation length of less than 20 minutes were associated with a low quality of care (OR for a waiting time between 15 and 60 minutes was 1.81 and for more than 60 minutes it was 3.20). No improvement of health status after consultation was also associated with low quality rating (OR of 2.93 for all users). Other key factors which determined the perception of an overall low quality of care were: bad health status at the time of the survey, acute and chronic diseases as the reason of seeking care in comparison to prevention, HSUs younger than 60 years, payment for consultation and not receiving medicines. Sex, education and socio-economic level were not found to be associated with low quality ratings in the model which included the institution as variable.

**Table 2:** Association between the overall perception of low quality of care and health service users' (HSU) characteristics

Variable		n (7,440)	Unadjusted OR (95% IC)	Adjusted OR (95% IC)
Sex	Female	4,734 (63.6%)	1.15 (0.93 - 1.43)	1.02 (0.82 - 1.28)

Age (years)	0-5	201 (2.7%)	0.84 (0.49 - 1.45)	1.07 (0.58 - 1.96)
	
	6-17	1,544 (20.8%)	1.05 (0.79 - 1.39)	1.61 (1.15 - 2.26)**
	
	18 -35	1,379 (18.5%)	1.24 (0.93 - 1.66)	1.72 (1.21 - 2.47)**
	
	36-47	1,309 (17.6%)	1.12 (0.84 - 1.50)	1.44 (1.05-1.98)*
	
	48-59	1,250 (16.8%)	1.20 (0.90 - 1.60)	1.35 (0.96 - 1.89)
	
	> = 60	1,757 (23.6%)	1	1

Schooling	None	1,196 (16.1%)	0.91 (0.69 - 1.20)	1.31 (0.89 - 1.93)
	
	Elementary	3,612 (48.6%)	0.94 (0.75 - 1.18)	1.08 (0.82 - 1.42)
	
	Secondary or higher	2,632 (35.4%)	1	1

Socioeconomic Status	0	2,884 (38.8%)	1.32 (0.95 - 1.83)	1.38 (0.93 - 2.03)
	
	1	1,561 (20.9%)	1.04 (0.73 - 1.47)	1.07 (0.70 - 1.62)
	
	2	2,188 (29.4%)	1.32 (0.95 - 1.83)	1.36 (0.94 -1.99)
	
	3	807 (10.9%)	1	1

Health status at the time of the survey	Bad	971 (13.1%)	1.97 (1.47 - 2.66)**	1.84 (1.32 - 2.56)**
	
	Regular	3,226 (43.4%)	1.64 (1.34 - 2.00)**	1.63 (1.31 - 2.03)**
	
	Good	3,243 (43.6%)	1	1

Reason for health service use	Acute care	4,420 (59.4%)	1.09 (0.75 - 1.58)	1.66 (1.11 - 2.48)*
	
	Chronic care	2,359 (31.7%)	1.13 (0.76 - 1.69)	1.28 (0.84 - 1.93)
	
	Preventative services	661 (8.9%)	1	1

No improvement in health status after consultation		1,518 (20.4%)	3.24 (2.62 - 4.00)**	2.93 (2.29 - 3.76)**

Waiting times (minutes)	<15	2,157 (29.0%)	1	1
	
	15 -60	3,242 (43.6%)	2.28 (1.69 - 3.08)**	1.81 (1.35 - 2.45)**
	
	>60	2,041 (27.4%)	4.94 (3.61 - 6.76)**	3.20 (2.35 - 4.35)**

Consultation length	<15	3,320 (44.6%)	1	1
	
	15-20	1,793 (24.1%)	2.36 (1.87 - 2.97)**	2.03 (1.60 - 2.57)**
	
	>20	2,327 (31.3%)	1.35 (1.02 - 1.79)*	1.19 (0.89 - 1.59)**

Payment for consultation (Yes)		2,791 (37.5%)	0.50 (0.40 - 0.62)**	1.42 (1.03 - 1.96)*

Prescription filling (No)		5,093 (68.5%)	0.85 (0.67 - 1.06)	1.32 (1,03 - 1.70)*

Institution	Private	2,189 (29.4%)	1	1
	
	Public	2,654 (35.7%)	2.93 (2.15 - 4.00)**	2.38 (1.63 - 3.47)**
	
	Social Security	2,597 (34.9%)	4.18 (3.17 - 5.52)**	4.36 (2.95 - 6.44)**

*significant at 5%; ** significant at 1%

According to the institution type providing care, common factors associated with low quality of care were consultation length of less than 20 minutes, no improvement in health status and reporting bad health status at the time of the survey (Table 3). Important differences of factors associated with quality of care between the types of institutions are: prescription filling was associated only with low quality rating in the case of HSUs of MOH. Education level was only associated in the case of HSUs of PrivI (the HSUs with less education more frequently perceive quality of care as low) whereas socio-economic level (HSUs with medium to high level perceived quality of care lower than those HSU with lower socio-economic level) and reasons for health services use (HSUs with acute events were more likely to perceive quality as low versus those receiving preventative services) which were only associated with HSUs of SSI.

**Table 3:** Multivariable binominal logistic regression model of factors associated with low quality of care ratings by health service users

Variable		Public Services OR (95% IC)	Social Security OR (95% IC)	Private Services OR (95% IC)
Sex	Female	0.93 (0.66 - 1.31)	1.13 (0.83 - 1.53)	1.07 (0.65 - 1.76)
Age	0-5	1.48 (0.61 - 3.58)	1.07 (0.28 - 4.16)	0.41 (0.09 - 1.78)
	6-17	1.69 (0.93 - 3.04)	1.48 (0.94 - 2.32)	1.49 (0.64 - 3.46)
	18 -35	1.70 (0.93 - 3.11)	1.23 (0.77 - 1.99)	3.09 (1.21 - 7.84)
	36-47	1.61 (0.91 - 2.84)	1.34 (0.86 - 2.08)	1.13 (0.51 - 2.47)
	48-59	1.22 (0.62 - 2.42)	1.18 (0.79 - 1.77)	1.63 (0.81 - 3.28)
Schooling	None	1.36 (0.70 - 2.64)	0.74 (0.44 - 1.25)	4.21 (1.90 - 9.30)**
	Elementary	1.05 (0.63 - 1.75)	0.79 (0.55 - 1.13)	2.88 (1.45 - 5.73)**
	Secondary or higher	1.0		
Socioeconomic status	0	2.34 (0.71 - 7.65)	1.48 (0.89 - 2.45)	0.72 (0.28 - 1.83)
	1	1.36 (0.41 - 4.51)	1.22 (0.74 - 2.01)	0.91 (0.35 - 2.35)
	2	2.58 (0.79 - 8.35)	1.56 (1.01 - 2.42)*	0.58 (0.22 - 1.50)
	3	1.0		
Health status at the time of the survey	Bad	1.85 (1.04 - 3.29)*	1.59 (1.03 - 2.46)*	2.66 (1.17 - 6.06)*
	Regular	1.75 (1.20 - 2.56)**	1.42 (1.02 - 1.99)*	1.76 (1.01 - 3.07)*
	Good	1.0		
Reason for health service use	Acute care	1.36 (0.66 - 2.79)	1.74 (1.05 - 2.89)*	1.38 (0.48 - 3.98)
	Chronic care	1.17 (0.53 - 2.56)	1.39 (0.84 - 2.30)	0.83 (0.28 - 2.51)
	Preventative services	1.0		
No improvement in health status after consultation		2.14 (1.47 - 3.12)**	2.93 (2.17 - 3.95)**	5.64 (3.19 - 9.97)*
Waiting times (minutes)	<15	1.0		
	15-60	1.96 (1.20 - 3.21)**	2.11 (1.33 - 3.34)**	1.24 (0.70 - 2.18)
	>60	3.29 (2.04 - 5.30)**	4.10 (2.53 - 6.63)**	1.41 (0.58 - 3.41)
Consultation length	<15	1.0		
	15-20	1.98 (1.34 - 2.93)	1.71 (1.21 - 2.41)*	2.65 (1.50 - 4.71)**
	>20	1.40 (0.89 - 2.20)**	1.14 (0.74 - 1.76)**	0.77 (0.42 - 1.41)**
Payment for consultation (Yes)		1.31 (0.91 - 1.88)	1.25 (0.57 - 2.71)	1.43 (0.73 - 2.79)
Prescription filling (No)		2.04 (1.45 - 2.88)*	1.26 (0.80 - 1.98)	0.66 (0.40 - 1.08)

*significant at 5%; ** significant at 1%

## Discussion

The results of the study contribute to existing knowledge about health care quality in several ways. In Latin America, including Mexico, there is a paucity of analysis of the HSUs' experience of health care *in combination *with their respective ratings as well as the definition of reference points of acceptable care using HSUs' own experiences. The results of this work indicate that defining reference points according to the HSUs' own perception of care is useful for setting targets for quality improvements. The results also show that prioritizing reduced waiting times, improved health status after consultation and increased consultation time would increase overall quality of care ratings.

Before discussing these results in more detail it is relevant to mention the study limitations. ENSANUT does not include aspects related to physician-patient communication, shared decision-making, selection of provider, the attitude of the health professionals and the time it takes to obtain an appointment with a physician. These aspects have been identified as relevant when analyzing perception of health care quality [25] and should be included in future National Health Survey instruments. Another limitation is that the survey does not permit measuring quality over time for the same HSUs; literature has shown that ratings change over time [26]. The sample size of HSUs in ENSANUT 2006 is not large enough to make meaningful comparisons between different Mexican states (Mexico has 32 states) although this would be very relevant as states are largely autonomous with respect to health service delivery. Finally, due to the fact that we analyzed reported experience of HSUs the reported travel, waiting and consultation time were estimated and frequently rounded (instead of 12 minutes the HSU rounded it downwards to 10 minutes and instead of 14 minutes 15 minutes were reported) which resulted in some inaccuracies.

Defining reference points using the quality ratings of HSUs could be very useful for quality improvement programs such as SICalidad in Mexico or in other countries as it quantifies how much travel and waiting times or expenditure need to be reduced to improve the general HSUs' perception of quality of care. Strength of the study is that it asked HSU about actual and not hypothetical cases of health service use. For instance, our results highlight that on average a travel and waiting time of 10 minutes respectively is acceptable to ambulatory HSUs of all institutions. However, the gap between the reference points and reported waiting time is largest for HSUs of SSI at 30 minutes and second for MOH at 20 minutes. Although the Ministry of Health quality program periodically reports waiting times it does not provide a measure regarding what waiting time HSUs on average have rated as acceptable [16]. In general, expenditure on medicines, laboratory tests and consultations were much higher than what were defined as acceptable expenditure by HSUs. Particularly, the expenditure on medicines, laboratory and consultation is very likely a barrier to access for HSUs of MOH since the large majority belongs to the lowest socio-economic group.

The reference points of acceptable care were defined for those aspects of care for which the survey instrument provided both types of information: HSU experience and their respective ratings. However, it would be very important in future surveys to include additional questions which allow combining reported experience with HSUs' ratings for other aspects related to quality of care (for example consultation length in the present instrument is only analyzed by asking HSU about the estimated duration; their respective ratings are omitted).

Second, the results show that the experiences of HSUs in access and use differed significantly between institution types which mean that quality improvement programs should take into account these differences and be tailored towards those aspects where quality is rated particularly low in each institution. For instance, although state-run institutions have committed themselves to provide medicines and laboratory test free of charge or for a small fee, [22] fewer HSUs of MOH received a prescription of medicines and laboratory tests compared to HSUs of SSI. Improving provision of medicines and laboratory tests is clearly an area of improvement for MOH. The long waiting times most frequently reported by HSUs of SSI in comparison to the other two institutions studied means that quality improvement program should develop strategies to reduce waiting time particularly in SSI.

Finally, our results help to prioritize which aspects of care need to be improved by showing which factors have the largest impact on HSU perception of low quality of care. In our study, institution was one of the key factors. This is in line with other authors who also found that the type of institution is the most important factor in terming overall perception of quality of care in Mexico [17,18]. It also is consistent with other studies which reported that prepaid services were rated lower in quality than fee-for-service visits [24]. Waiting times and no improvement in health status after consultation were other factors. That HSUs of SSI most often reported no improvement in health status after consultation seems contrary to the finding that more SSI HSUs receive medicines, laboratory tests and consultation free at the point of care than their counterpart at MOH and PrivI. The question of which factors contributed to less beneficial health outcomes for HSUs of SSI -even though material supplies such as medicines and laboratory facilities were available- is certainly very relevant and needs further investigation. Particularly, one must consider that 32% of those HSUs with social security decided to use PrivI, which results in large inefficiencies where households which are already paying social security insurance incur out-of-pocket expenditure for private consultations. We explored if the perception of improved health status after health service use could have a mediating effect on other variable, but no significant associations with other variables were found. We also examined whether the institution type was a confounding factor or a mediating factor. In theory, institution could be a confounding factor since it is related with the socioeconomic status and education level, both of which are associated with the perception of quality of care. However, the association of the variables remained the same when conducting models for each institution type.

Our results show that the length of the consultation was another important factor influencing overall rating of quality of care, where duration of less than 20 minutes was associated with lower quality of care rating. The saturation of SSI services could be one explanation for the shorter duration compared to MOH and PrivI [27]. Consultation lengths vary by countries and by type of services: in Germany, average consultation time was 7.4 minutes, in Belgium 15 minutes and in India 5 minutes [27,28]. The observed differences between MOH and SSI on one side and PrivI on the other are probably related to the different organization of care where private physicians need to invest time to ensure that the patient returns [27]. Since our results show that consultation length was relevant in the overall rating of quality it should be included in the indicators of quality improvement programs such as SICalidad.

Other authors have generally found that older people, women, those of lower socio-economic status and better health status are associated with high ratings of quality of care [29]. This is in line with our results which show that HSUs with better health status and older than 35 years rated quality higher in comparison to other groups of HSUs. However, in the case of HSUs of SSI we found that - contrary to the other previous findings- medium/high socio-economic status was associated with lower ratings of quality of care in comparison to those HSUs of high socio-economic level. Sex was not associated with quality rating.

## Conclusions

As strategies to improve health care quality have been important elements of health care reforms in Latin America in the last decades [30] evaluation of the impact of the reforms and justifying increasing expenditure in health measuring quality of care have become increasingly relevant. Quality of care can be measured in various ways and it has been argued that HSU perception is an essential component, since HSUs have the right to a certain standard of quality of care and because the perception of high quality improves their health outcomes [31]. This study shows that the combination of factual information of the HSU experience with their perception is important to identify how services needs to change in order improve HSU perception of its quality. The results of this study also help to prioritize which aspects of health care quality are more relevant to HSU. The 2006 National Health Survey instruments in Mexico allowed only defining reference points for travel and waiting time as well as expenditure on medicines, laboratory and consultation. Establishing reference points for other aspects related to quality of care will be relevant for monitoring quality in health services.

## Competing interests

The authors declare that they have no competing interests.

## Authors' contributions

ALS analyzed the data with support of SKE, VJW and YSAT. ALS wrote the first draft of the methods and result section of the manuscript which was substantially revised by VJW and SKE. The introduction, discussion and final version of the manuscript was prepared by VJW and revised by all authors.

## Pre-publication history

The pre-publication history for this paper can be accessed here:

http://www.biomedcentral.com/1472-6963/10/178/prepub
